# Dried Plums, Prunes and Bone Health: A Comprehensive Review

**DOI:** 10.3390/nu9040401

**Published:** 2017-04-19

**Authors:** Taylor C. Wallace

**Affiliations:** 1 Department of Nutrition and Food Studies, George Mason University, Fairfax, VA 22030, USA; taylor.wallace@me.com; Tel.: +1-270-839-1776; 2 Think Healthy Group, Inc., Washington, DC 20001, USA

**Keywords:** dried plum, prune, bone

## Abstract

The 2015–2020 Dietary Guidelines for Americans advocate for increasing fruit intake and replacing energy-dense foods with those that are nutrient-dense. Nutrition across the lifespan is pivotal for the healthy development and maintenance of bone. The National Osteoporosis Foundation estimates that over half of Americans age 50+ have either osteoporosis or low bone mass. Dried plums, also commonly referred to as prunes, have a unique nutrient and dietary bioactive profile and are suggested to exert beneficial effects on bone. To further elucidate and summarize the potential mechanisms and effects of dried plums on bone health, a comprehensive review of the scientific literature was conducted. The PubMed database was searched through 24 January 2017 for all cell, animal, population and clinical studies that examined the effects of dried plums and/or extracts of the former on markers of bone health. Twenty-four studies were included in the review and summarized in table form. The beneficial effects of dried plums on bone health may be in part due to the variety of phenolics present in the fruit. Animal and cell studies suggest that dried plums and/or their extracts enhance bone formation and inhibit bone resorption through their actions on cell signaling pathways that influence osteoblast and osteoclast differentiation. These studies are consistent with clinical studies that show that dried plums may exert beneficial effects on bone mineral density (BMD). Long-term prospective cohort studies using fractures and BMD as primary endpoints are needed to confirm the effects of smaller clinical, animal and mechanistic studies. Clinical and prospective cohort studies in men are also needed, since they represent roughly 29% of fractures, and likewise, diverse race and ethnic groups. No adverse effects were noted among any of the studies included in this comprehensive review. While the data are not completely consistent, this review suggests that postmenopausal women may safely consume dried plums as part of their fruit intake recommendations given their potential to have protective effects on bone loss.

## 1. Introduction

The 2015–2020 Dietary Guidelines for Americans (DGA) advocate for healthy eating patterns that include a variety of fruits. This includes all fresh, frozen, canned and dried fruits and fruit juices [1]. The recommended intake of fruit in the Healthy US-Style Eating Pattern at the 2000-kcal level is two cup-equivalents of fruit per day. Increasing the amount and variety of fruits Americans consume is a strategy that helps individuals meet a wide range of nutrient requirements. However, per the 2015–2020 DGA, average intake of fruit is well below recommendations for almost all age-sex groups, except in children ages 1–8 years [1]. Average intake of fruit is lowest among girls ages 14–18 years and in women age 51+ years [1], two critical time points in bone development and maintenance.

Osteoporosis is a rising public health concern, given the aging population and suboptimal dietary intakes of dairy, fruits, vegetables and whole grains, which provide a variety of essential nutrients that influence bone accretion and maintenance across the lifespan. The National Osteoporosis Foundation estimates that 10.3% of Americans over the age of 50 years have osteoporosis (*t*-score ≤ 2.5), and 43.9% have low bone mass (also commonly referred to as osteopenia; *t*-score ≤ 1.0), a risk factor for osteoporosis [2]. The risk of fractures increases with age among individuals age 50+, and differs by sex, race and ethnicity [2,3]. Although many factors contribute to this debilitating event, the most significant causes are reduction in bone mass, structural deterioration and increased frequency of falls. It 2005, it was estimated that the over two million incident osteoporotic fractures occurring annually in the U.S. had an economic burden of $16.9 billion, which is anticipated grow to three million fractures at a cost of $25.3 billion by 2025 [4]. Men account for 29% of these fractures and 25% of the cost burden [4]. Optimization of lifestyle factors known to influence bone mass and strength is an important strategy aimed at reducing the risk of fractures later in life.

Plums are a type of drupe fruit that belong to the subgenus *Prunus* (family Rosaceae). They differ from other subgenera of drupe fruits (cherries, peaches, etc.) since the shoots have a terminal bud and unclustered single side buds, flowers combine in groups of one to five on short stems, the fruit has a crease running down one side and a smooth seed. There are over 40 species of plums currently documented, although two species, the European plum (*Prunus domestica*) and Japanese plum (*Prunus salicina* and hybrids) are of commercial significance globally [5]. The origin of European plum is thought to have been near the Caspian Sea, while Japanese plums originated in China, but derived their name from the country where they were cultivated. European plums were introduced in the U.S. by pilgrims in the 17th century, while Japanese plums were introduced to the U.S. in the late 19th century. China, Serbia and Romania are the world’s leading producers of plums. Worldwide, greater than 11.2 million metric tons of plums were harvested in 2014 per the Food and Agriculture Organization (FAO) of the United Nations [6]. While all prunes originate from fresh plums, not all plum varieties are considered prunes. Commercialized prunes, also commonly known as dried plums, are the dehydrated version of the cultivar *Prunus domestica* L. cv d’Agen. This specific variety has a naturally-occurring sugar content that enables it to be dried while still containing the pit, without being fermented. The State of California produces ~99% of the plums in the U.S. and ~40% of the world’s dried plums [7]. 

Dried plums are widely known for their laxative effect, which is commonly attributed to their dietary fiber content [8], but is also likely influenced by the significant amounts of phenolics (e.g., chlorogenic acid) and sorbitol present in the fruit. Dried plums are not only a source of dietary fiber, but also a good source of potassium and vitamin K (Table 1). One serving or ~4 dried plums is 92 kilocalories and provides 2.4 g of dietary fiber, 280 mg of potassium and 22.8 µg of vitamin K. Dried plums also contain several dietary bioactives, including phenolic compounds, such as 3-caffeoylquinic acid, 4-caffeoylquinic acid, 5-caffeoylquinic acid, 3-p-coumarolylquinic acid, caffeic acid, p-coumaric acid and quercetin-3-*O*-rutinoside [9], whose benefits may extend beyond the basic nutrition requirements of humans. There is an emerging body of evidence from laboratory, animal and human studies that suggests that dried plums may exert an effect on bone health. Hooshmand and others found that two servings (100 g) of dried plums per day slowed the rate of bone turnover and helped to improve bone mineral density (BMD) in a clinical study of 160 randomized postmenopausal women (100 completed the study) not receiving hormone replacement therapy [10]. A more recent clinical study by the same group confirmed the bone protective effects in postmenopausal women receiving one serving of dried plums per day [11]. However, a comprehensive review of dried plums and bone health is not currently present in the peer-reviewed scientific literature.

## 2. Methods

### 2.1. Literature Search

A comprehensive literature search was conducted as of 24 January 2017 using the PubMed database. The search methodology is outlined in Table 2. A systematic literature search was not conducted for lack of clinical and observational evidence and since the focus was to evaluate potential mechanisms from various types of data.

Included in the review were cell, animal, population and clinical studies in the English language that assessed the effects of dried plums or extracts of the former on markers of bone health. All articles were screened by title/abstract and, in some cases, full-text. A complete manual search of reference lists of original studies was also conducted. Excluded studies (*n* = 26) were those of any kind that did not assess dried plum (prune) or plum intake on one or more markers or clinical endpoints of bone health.

### 2.2. Data Extraction

Quantitative and qualitative data information from each study, including author and year of study, geographic study location, study design, product information, intervention, population, markers measured, duration and results, were extracted (Table 3, Table 4 and Table 5).

## 3. Results

The literature search of the PubMed database yielded 50 articles [10,11,12,13,14,15,16,17,18,19,20,21,22,23,24,25,26,27,28,29,30,31,32,33,34,35,36,37,38,39,40,41,42,43,44,45,46,47,48,49,50,51,52,53,54,55,56,57,58,59,60,61]. After title/abstract review, 22 articles were screened in full-text and included in this comprehensive review [10,11,12,13,14,15,16,17,18,19,20,21,22,23,24,25,26,27,28,29,31,32]. Two additional studies [30,33] were included after examination of the reference lists of the 22 studies identified in the literature search. Data and results from each of the 24 included studies are listed in Table 3, Table 4 and Table 5.

Four clinical trials were identified in this comprehensive review [10,11,12,13,14]. Most the studies retrieved for full-text review were animal studies involving either rats or mice (16 total) [15,16,17,18,19,20,21,22,23,24,25,26,27,28,29,30], although three (cell) studies were also identified [31,32,33]. No observational studies were identified in the PubMed literature search or after examination of the reference lists of included studies.

## 4. Discussion

Dried plums are being increasingly recognized for their role in bone health. This comprehensive review supports that consumption of dried plums is safe and may be a bone healthy option for postmenopausal women wishing to satisfy daily requirement for fruit as outlined by the 2015–2020 DGA. It is important to note that dried plums contain a higher amount of vitamin K as compared to other commonly-consumed fruits, which may influence bone health by helping to improve calcium balance.

The quality of clinical studies included in this comprehensive review was acceptable, noting that none utilized a sample size based on a priori power calculation, nor were the treatment allocations able to be concealed from the participants and/or investigators. All four clinical studies identified were derived from the same laboratory group [10,11,12,13,14], meriting the need for replication by additional investigators. Clinical studies included in this review had several limitations, such as a short duration of 3–12 months, which is a narrow window to see significant changes in BMD. All four clinical studies were un-blinded, and only the Hooshmand et al. 2016 [11] had an inactive placebo group. The Hooshmand et al. 2011 [10] and Hooshmand et al. 2014 [13] manuscripts used dried apples as the control group and represent the same study population with additional biomarkers being measured for the latter publication post hoc (BMD data are presented twice). Participants in this study also received 500 mg of calcium and 400 IU of vitamin D during the intervention [10,13], even though administration of these supplements was equal across both arms. Simonavice et al., 2014 [14], assessed the effects of dried plums and resistance training vs. resistance training alone on blood markers of bone and inflammation in female breast cancer survivors. While this study found null effects, these results are likely not generalizable to healthy postmenopausal women experiencing normal hormone-related bone loss. Resistance training has been shown to have a larger effect on preventing bone loss as compared to most dietary interventions and could have masked the much smaller the effects, if present, exerted by dried plums. The Hooshmand et al., 2016 [11], found that dried plum consumption at 50–100 g/day for a period of six months prevented loss of total body BMD, but not spine, hip or ulna BMD, likely due to its shorter duration.

Consistent improvements in BMD at several sites were noted in animal studies designed to model conditions at or before peak bone mass, pregnancy, post-menopause, osteopenia and/or osteoporosis. Rat models of ovarian hormone deficiency have been used for over 25 years to simulate postmenopausal bone loss in humans. Ovarian hormone deficient rats and postmenopausal women have many similar characteristics when it comes to bone loss. These characteristics include increased rates of bone turnover with resorption exceeding formation, an initial rapid phase of bone loss followed by a slower phase due to the ovariectomy, greater loss of trabecular vs. cortical bone, decreased intestinal absorption of calcium and a similar response to drug (e.g., bisphosphonate therapy) and lifestyle interventions (e.g., physical activity) [41]. Indeed, animal studies show that dried plums and/or their polyphenol-rich extracts can beneficially affect both BMD and bone biomarkers. The animal and cell studies presented in this comprehensive review are consistent with and supportive of the theory that a diet high in phenolics and/or flavonoids may enhance bone formation and inhibit bone resorption through their actions on cell signaling pathways that influence osteoblast and osteoclast differentiation [62]. Total body BMD and BMD at specific sites, as well as several blood biomarkers, including AP, BAP, BSAP, OPG, RANKL and TRAP-5b, have been shown to be consistently and beneficially impacted across both clinical and animal studies. Animal studies also collectively support that dried plums may beneficially influence bone area and micro-architecture.

Several bone turnover markers seemed to be improved among clinical studies; however, there was a lack of consistency among many of the markers across and between both clinical and animal studies. For instance, Arjmandi et al., 2002 [12], found significant increases in bone alkaline phosphatase (BAP), but the latter larger study Hooshmand et al., 2011 [10], reported a decrease in BAP. Noting the abundance of bone turnover markers measured in both research and the clinical setting, the International Osteoporosis Foundation (IOF) and International Federation of Clinical Chemistry and Laboratory Medicine (IFCC) Bone Markers Working group recently reviewed the scientific literature to determine the clinical potential of bone turnover markers, which includes the prediction of fracture risk and monitoring treatments for osteoporosis [63]. The IOF/IFCC working group identified one bone resorption marker (s-CTX, serum C-terminal telopeptide of type I collagen) and one bone formation marker (s-PINP, serum procollagen type 1 N propeptide) to be used as reference markers and measured by standardized assays in observational and intervention studies [63]. While only one animal study assessed the effects of dried plums on CTX [27] and two on PINP [27,28], collectively, the animal studies included in this review showed beneficial effects of dried plums and/or their polyphenol-rich extracts on most, but not all, markers of bone turnover. Nevertheless, bone turnover markers are likely too premature in their standardization and clinical utility to accurately predict small changes in bone, as expected in dietary interventions. Differences in study design, dose and duration may also contribute to the inconsistencies in the bone turnover markers measured across and between rodent and cell studies.

### Future Research

Identification of the active components, particularly individual phenolics, and their potential modes of action are necessary to fully understand the overall effect of dried plums on bone health across the lifespan. While existing data indicate that consumption of dried plums may be beneficial in postmenopausal women with ongoing bone loss, future clinical and prospective cohort studies in premenopausal women, men and adolescents prior to peak bone mass accrual are necessary to confirm their effects across the population and to make generalizable dietary guidance statements.

Recent epidemiological studies show that phenolic compounds may have a stronger association with bone than general fruit and vegetable consumption [62]. Even though BMD is a validated biomarker of bone health, fractures represent the most significant clinical endpoint of bone health across the lifespan. Prospective cohort studies designed to assess potential associations of dried plum intake on both fracture risk and changes in BMD across the population and various subpopulations are greatly needed to confirm the findings of studies included in this comprehensive review.

## 5. Conclusions

Dried plums are an easy means to help individuals meet their daily recommendations for fruit intake. The beneficial effects of dried plums on bone health may be in part due to the unique variety of phenolics and nutrients present in the fruit. Animal and cell studies suggest that dried plums and/or their extracts enhance bone formation and inhibit bone resorption through their actions on cell signaling pathways that influence osteoblast and osteoclast differentiation; however, results on specific markers are not consistent across and between studies. Animal studies are somewhat consistent with small clinical interventions that show dried plums may exert beneficial effects on total body and site-specific BMD. Long-term prospective cohort studies using fractures and BMD as primary endpoints are needed to confirm the effects of smaller clinical, animal and mechanistic studies. No adverse effects were noted among any of the studies included in this comprehensive review. While the data are not completely consistent, this review suggests that postmenopausal women may safely consume dried plums as part of their fruit intake recommendations given their potential to have protective effects on bone loss.

## Figures and Tables

**Table 1:** Nutritional profile of dried plums per 100 g.

Nutrient	Unit	DV	Plums, Dried (Prunes) (09291) ^a^
Macronutrients			
Water	g	ND	30.92
Energy	Kcal	2000	240
Protein	g	50	2.18
Fat	g	78	0.38
Carbohydrate	g	275	63.88
Fiber	g	28	7.1
Minerals			
Calcium	mg	1300	43
Iron	mg	18	0.93
Magnesium	mg	400	41
Phosphorus	mg	1000	69
Potassium	mg	4700	732
Sodium	mg	2300	2.0
Zinc	mg	15	0.44
Copper	mg	2	0.281
Manganese	mg	2	0.299
Selenium	μg	70	0.3
Vitamins			
Vitamin C	mg	60	0.6
Thiamin	mg	1.5	0.51
Riboflavin	mg	1.7	0.186
Niacin	mg	20	1.882
Pantothenic acid	mg	10	0.422
Vitamin B6	mg	2	0.205
Folate	μg	400	4.0
Choline	mg	550	10.1
Vitamin B12	μg	6	0.0
Vitamin A	IU	5000	781
Vitamin D	μg	20	0.0
Vitamin E	mg	30	0.43
Vitamin K	μg	80	59.5

^a^ Nutrient Database Number (NDB No.) in the USDA Food Composition Databases. DV = daily value; ND = not defined by FDA.

**Table 2:** Search strategy.

Search No.	Search	Results	Search Type
1	bone and bones (MeSH Terms)	536,127	Advanced
2	bone AND (fracture* OR density OR resorption OR development)	340,163	Advanced
3	osteoporosis	73,977	Advanced
4	osteoblasts	36,771	Advanced
5	osteoclasts	19,477	Advanced
6	#1 OR #2 OR #3 OR #4 OR #5	762,438	Advanced
7	Prune(TIAB) OR plum(TIAB) OR dried plum(tiab)	2460	Advanced
8	#6 AND #7	50	

MeSH = medical subject heading; TIAB = title/abstract.

**Table 3:** Clinical trials.

Reference	Location	Design	Plum Product	Intervention	Population	Markers Measured	Duration	Results
Arjmandi et al. 2002 [12]	USA	RCT	Dried plum (*P. domestica*)	100 g/day DP or 75 g/day dried apple	Postmenopausal women (*n* = 58)	IFG-I, IGFBP-3, AP, BSAP, TRAP, phosphorus, magnesium, calcium, urine-DPD, urine-HP	3 months	DP led to borderline significant increases in AP and IGF-1. Borderline significant increase in BSAP No significant differences on other markers measured.
Hooshmand et al. 2011 [10]; Hooshmand et al. 2014 [13]	USA	RCT	Dried plum (*P. domestica*)	100 g/day DP or 75 g/day dried apple	Postmenopausal women with osteopenia (*n* = 160 enrolled; 100 completed)	BMD (spine, ulna, total hip and whole body), RANKL, OPG, sclerostin, osteocalcin, TRAP-5b, BALP, DPD, phosphorus, calcium	1 year	Significant increase in BMD at the spine and ulna in both groups, however increases were significantly greater in the DP group compared to dried apple control. Borderline significant increase in RANKL and OPG on DP group. Significant decrease in sclerostin, BSAP and TRAP-5b. No significant differences on other markers measured.
Simonthsnavice et al. 2014 [14]	USA	Intervention	Dried plum (*P. domestica*)	90 g/day DP with combination resistance training vs. resistance training alone.	Female breast cancer survivors (*n* = 23)	BMD (lumbar spine, femur and forearm), TRAP-5b, BSAP, CRP	6 months	No significant differences between groups or any group-by-time interaction.
Hooshmand et al. 2016 [11]	USA	RCT	Dried plum (*P. domestica*)	0, 50 g/day or 100 g/day DP.	Postmenopausal women (*n* = 48)	BMD (total body, total hip, L1-L4 and ulna), BAP, TRAP-5b, BAP/TRAP-5b ratio, hs-CRP, IGF-1, sclerostin, RANKL, OPG, RANKL/OPG ratio, 25(OH)D, calcium, phosphorus	6 months	Compared to controls: Both doses of DP prevented loss of total body BMD but not hip, spine or ulna BMD as compared to the control group. TRAP-5b decreased at 3 months and this was sustained at 6 months in both 50 and 100 g DP groups. BAP/TRAP-5b ratio was significantly greater at 6 months in both DP groups. No significant differences on other markers measured.

25(OH)D = 25-hydroxyvitamin D; AP = alkaline phosphatase; BAP = bone alkaline phosphatase; BMD = bone mineral density; BSAP = bone-specific alkaline phosphatase; DP = dried plums; DPD = deoxypyridinoline; HS = hydroxylysylpyridinoline; hs-CRP = high sensitivity C-reactive protein; IGF-1 = insulin-like growth factor-1; IGFBP-3 = insulin-like growth factor-binding protein-3; OPG = osteoprotegerin; RANKL = receptor activator of nuclear factor kappa-B ligand; TRAP = tartrate-resistant acid phosphatase; TRAP-5b = tartrate-resistant acid phosphatase-5b.

**Table 4:** Animal studies.

Reference	Location	Animal Model	Plum Product	Methods	Markers Measured	Duration	Results
Arjmandi et al. 2010 [15]	USA	Sprague-Dawley rats	Dried plum (*P. domestica*), DP puree, DP juice, DP pulp/skin, DPP	After surgery to establish bone loss, rats placed on various diets supplemented with 13 different combinations of fructooligosaccharides and DP vs. a control diet.	BMD and BMC (whole body, right femur, 4th lumbar vertebrae), calcium loss (4th lumbar), TbS, serum OC, serum IGF-1, calcium, phosphorus, and magnesium.	60 days	Compared to the other treatments, diets supplemented with 5% FOS and 7.5% DP was most effective in reversing both right femur and fourth lumbar BMD and fourth lumbar calcium loss while significantly decreasing TbS. No significant effects of treatment on serum or urine measures of bone turnover.
Bu et al. 2007 [16]	USA	Male Sprague-Dawley rats	Dried plum (*P. domestica*) vs. parathyroid hormone	Diet supplementation of 6-month old male rats with 25% DP vs. a control diet.	BMA, BMC, BMD (whole body, femur, vertebrae), trabecular architecture, cortical bone, serum ALP, serum protein, BV/TV, TbN, TbSp, femur and vertebral (connectivity density, SMI, linear attenuation), total force, stiffness, physiological force.	90 days	DPs induced a significant increase in vertebra and femoral BMD compared to controls. DPs induced a significant increase in femur BMC compared to controls. The DP group had significantly: Higher femur and vertebra BV/TV, TbN. Higher femur connectivity density, femur and vertebral linear attenuation. Higher cortical thickness and cortical area. Lower TbSp and femur SMI, Higher total force, stiffness, and physiological force. Lower average von Mises stresses.
Deyhim et al. 2005 [17]	USA	Sprague-Dawley rats	Dried plum (*P. domestica*)	Dietary supplementation of adult osteopenic rats with 5%, 15% or 25% DP vs. a control diet.	Serum ALP, TRAP activities, calcium, magnesium, IGF-I, BMD (femur, tibia, vertebra), trabecular microarchitecture, urinary DPD, L4 BMD, BV/TV, connectivity density, TbSp, and TbTh.	60 days	Compared to OVX controls: All DP groups had significantly higher femur BMD, tibia BMD, as well as lower TbSp. 25% DP groups had significantly higher L4 BMD, BV/TV, and connectivity density. 15% and 25% DP groups had significantly higher TbN and lower TbTh.
Franklin et al. 2006 [18]	USA	Male Sprague–Dawley rats	Dried plum (*P. domestica*)	Dietary supplementation of male rats with 5%, 15% or 25% DP vs. a control diet.	Whole body BMC, BMA, BMD), BMC (femur, L4 vertebra), trabecular bone microarchitecture markers (BV/TV, TbN, TbSp), serum ALP, osteocalcin, IGF-I, RANKL, OPG, cortical strength, cortical area, medullary area, cortical porosity, distal femur and L4 vertebral (SMI, connectivity density, LinAtt), IGF, DPD, OPG, RANKL.	90 days	15% and 25% DP groups significantly prevented a reduction in whole body BMD, as well as femur and L4 vertebra BMC. 15% and 25% DP groups protected against the decrease in mechanical strength required to break the femur bone. Compared to controls: 5% and 25% DP groups had significantly higher distal femur BV/TV. 25% DP group had significantly higher L4 vertebra BV/TV, TbN and significantly lower L4 vertebra TbSp. All DP groups had significantly higher distal femur TbN and lower distal femur TbSp, DPD, RANKL. 15% and 25% DP groups had significantly higher cortical strength and lower vertebral SMI and OPG. 25% DP group had significantly lower femur SMI and higher femur and vertebral connectivity density, vertebral LinnAtt, and IGF. 5% and 25% DP groups had significantly higher LinAtt. No significant differences on other markers measured.
Halloran et al. 2010 [19]	USA	Harlan Sprague Dawley mice	Dried plum (*P. domestica*)	Dietary supplementation of adult and old male mice with 15%, 25% DP vs. a control diet.	BV/TV, TbN, TbSp, P1NP, SMI, connective density, degree of anisotropy, ObS, OcS, BFR, cortical thickness, bone area, cortical area, Medullary area, BMD and PYD.	6 months	Within both adult and old mice, increasing DP supplementation was associated with greater BV. Mice fed 25% DP had significantly greater BV compared to controls. Mice fed 25% DP had significantly greater BV compared to those fed 15%. The differences in magnitude of the percent changes between control mice and those fed 25% DP were significantly greater in adult vs. old mice. Compared to controls: Adult mice fed 25%DP had higher BV/TV, TbN, connective density, and lower SMI. Old mice fed 25% DP had higher degree of anisotropy and cortical thickness, and lower medullary area and PYD. Old mice fed 15% DP had higher cortical area. No significant differences on other markers measured.
Johnson et al. 2011 [20]	USA	Sprague-Dawley rats	Dried plum (*P. domestica*)	Female ovarian hormone deficient rats a fed control, soy, soy + FOS, soy + 7.5% DP, and soy + 7.5% DP + FOS diet vs. a control diet.	BMD, BMC (Whole body, right femur, 4th lumbar vertebrae), serum ALP, urinary creatinine, urinary DPD, femur strength, TbN, BV/TV, TbTh, and TbSp.	60 days	Whole body and 4th lumbar BMD were significantly higher in diets with DP + FOS compared to the control and soy diets. No significant differences on other markers measured.
Leotoing et al. 2016 [21]	France	Wistar rats	High and low chlorogenic acid dried plum (*P. domestica*) and DP juice concentrate (15%)	Female rats High and low chlorogenic acid dried plum (*P. domestica*) and DP juice concentrate (15%) diets vs. a control diet.	Urinary DPD, OC, CPII, CTX-II, BMD (Total femoral, metaphyseal), BMC, urine calcium, primary pre-osteoblasts (proliferation, ALP), bone remodeling index, and cartilage remodeling index.	90 days (in vivo), 7 days (ex vivo)	10 and 50 μmol/L concentrations of neochlorogenic, chlorogenic, or caffeic acid significantly decreased pre-osteoblast ALP activity and increased pre-osteoblast proliferation. The low chlorogenic acid DP juice and DP juice concentrate groups showed significantly higher trabecular distal BMD, significantly increased cortical BMD, and increased total BMC compared to control. High chlorogenic acid DP juice group had significantly higher trabecular distal BMD compared to controls. High chlorogenic acid DP juice, low chlorogenic acid DP juice + fiber and low chlorogenic acid DP juice concentrate significantly prevented increase in OC. Low chlorogenic acid DP juice + fiber and low chlorogenic acid DP juice concentrate significantly prevented increase in DPD. Both high and low chlorogenic acid DP juice and DP juice concentrate lead to higher urinary calcium excretion compared to controls. Only high chlorogenic acid DP juice significantly counteracted the decrease in CPII. Only the high chlorogenic acid DP juice group had significantly higher CRI.
Monsefi et al. 2013 [22]	Iran	Pregnant mice	Dried plum (*P. domestica*) extract (8 mL/kg) and DP hydroalcoholic extracts (1.6 g/kg)	Pregnant mice were fed DP extracts vs. a control diet and outcomes measured on their fetuses.	Serum calcium, magnesium, ALP, bone calcium, and phosphorus.	30 days	Non-pregnant mice fed DP extract had significantly higher bone calcium compared to non-pregnant controls. Non-pregnant mice fed DP hydroalcoholic extracts had significantly higher bone phosphorus compared to non-pregnant controls. Non-pregnant mice fed both DP extract and DP hydroalcoholic extract had significantly higher bone calcium compared to non-pregnant controls.
Pawlowski et al. 2014 [23]	USA	Sprague-Dawley rats	Dried plum powder extract (0.20% and 0.45% *w*/*w* total dietary polyphenols)	Randomized, crossover intervention trial to evaluate 12 different polyphenolics containing diets on bone turnover.	Urine calcium (total and ^45^Ca), NTx and ALP.	10 days	Bone calcium retention was significantly improved due to dietary intervention with 0.45% DP extract compared to baseline. 0.45% DP extract improved bone calcium retention compared with the 0.20% DP extract. No significant effect on other outcomes.
Rendina et al. 2012 [24]	USA	Adult female C57BL/6J mice	Dried plum (*Prunus domestica*)	Adult female mice placed on 5%, 15% or 25% DP intervention vs. a control diet.	BMA, BMC and BMD of the 4th to 5th lumbar vertebrae (L4–L5), TbN, BV/TV, TbTh, TbSp, connectivity density, SMI, PINP IGF-I, NFATc, Runx2, biomechanical properties of trabecular bone, OC, IL-6, and TNF-α.	4 weeks	Mean BMC and BMA were significantly higher in the 25% DP group compared to the control. 15% DP group had a significantly higher plasma IGF-1 compared to the control. 15% and 25% DP groups significantly increased BV/TV compared to the control. 15% and 25% DP groups significantly decreased TbSp beyond that of the control group. 15% and 25% DP groups experienced a significant increase in vertebra TbTh compared to the control. 15% and 25% DP groups had significantly lower Von Mises stress distribution compared to the control. 15% and 25% DP groups had significantly higher vertebral connective density and tibia apparent mean/density, and lower vertebral SMI and OC expression and TNF-α. 25% DP group had significantly higher apparent mean/density and tibia connective density, and significantly lower tibia SMI. 25% DP group significantly increased TbN compared to the control. All doses of DP groups had significantly lower plasma PINP, NFATc and Runx2 compared to the control.
Rendina et al. 2013 [25]	USA	Adult osteopenic ovariectomized C57BL/6 mice	Dried plum (*Prunus domestica*), 25%	This study was designed to compare the efficacy of DP, apple, apricot, grape, and mango vs. a control in the restoration of bone in an osteopenic mouse model.	Whole body and L4–5 (BMA, BMC, BMD), TbN, BV/TV, TbTh, TbSp, SMI, biomechanical testing of vertebra and tibia, connective density, NFATc1, ALP, Col1a1, OC, Bak1, Casp3, and Casp9.	8 weeks	Compared to the control the DP group had significantly higher whole body and spine BMA, BMD and BMC. DP group had significantly higher vertebral BV/TV, TbN, TbTh, connective density, SMI, and trabecular density compared to the control group. DP group had significantly higher proximal tibia BV/TV compared to the control group. DP group had significantly higher vertebral total force, stiffness, size independent stiffness compared to the control group. DP group had significantly lower NFATc1 compared to the control group. DP group had significantly higher Bak1 and lower Casp3 and compared to the control group. No significant differences on other markers.
Schreurs et al. 2016 [26]	USA	Male C57BL/6J mice	Dried plum (*Prunus domestica*), 25%	This study randomized mice to 25% DP intervention vs. a control to protect from bone loss and then later exposed them to ionizing radiation.	Nfe2l2, RANL, MCP-1, OPG, TNF-α, TbN, BV/TV, TbTh, TbSp,	7–21 days	Compared to the irradiated controls, levels of Nfe2l2, RANKL, MCP-1, OPG, and TNF-α in the DP group were not statistically different. After exposure to radiation, DP mice did not have any significant decrease in TbN, BV/TV, TbTh or TbSp indicating a radio-protective effects against cancellous bone loss compared to irradiated controls. DP fed mice had significantly higher BV/TV, TbTh and TbN after being exposed to simulated space radiation compared to control diet.
Shahnazari et al. 2016 [27]	USA	C57Bl/6 mice	Dried plum (*Prunus domestica*)	Skeletally mature (6-month-old) and growing (1- and 2-month-old) male mice were placed on a 5%, 15% or 25% DP intervention vs. a control diet.	BV/TV, TbTh, TbN, SMI, OcS, ObS, MAR, MS/BS, BFR/BS, Ctsk, OPG, RANKL, CTX, and P1NP.	1–4 weeks	BV/TV and TbTh significantly increased and SMI significantly decreased after 2 and 4 weeks of DP. TbN significantly increased after 4 weeks of DP. After 2 and 4 weeks of DP: OcS, ObS, MAR, MS/BS, BFR/BS decreased significantly. Osteoclasts significantly decreased. DP fed mice had significantly lower: Ctsk gene expression. Immune-related cytokines (IL-1a, IL-1b, IL-10, IL-12, IL-13, IL-17, TNF-α, and MCP-1). CTX BV/TV increased significantly in mice fed 5%, 15% or 25% DP. TbTh increased significantly among mice fed 25% DP. TbN increased significantly in mice fed 5%, 15% or 25% DP. SMI decreased significantly for mice fed 15% and 25% DP. Among 2-month-old mice, ObS increased significantly among mice fed 5%, 15% or 25% DP. Among 3-month-old mice, ObS increased significantly among mice fed 5% DP. No significant differences on other markers.
Smith et al. 2014b [28]	USA	Female Sprague-Dawley rats	Dried plum (*Prunus domestica*)	Osteopenic rats were placed on 5%, 15% or 25% DP intervention vs. a control diet.	BMD (whole body, femur and vertebra), BV/TV, TbN, TbSp, connective density, TbTh (proximal tibia, vertebra), Cortical thickness, cortical area, medullary area, cortical porosity, DPD, P1NP, cancellous BFR and MS/BS, MAR, MS/bone area, BFR/BV, Periosteal (BFR, MS, MAR), endocortical (BFR, MS, MAR), Bmp2, Bmp4, Coll1a, IGF-1, Nfatc1, and RANKL.	6 weeks	Compared to controls: Vertebral BMD increased significantly in 15% and 25% DP groups Femur BMD increased significantly in 5%, 15%, and 25% DP groups Whole body BMD increased significantly in 5%, 15%, and 25% DP groups Within the vertebra and when compared to controls: BV/TV, TbN, TbSp, connective density increased significantly in 5%, 15% and 25% DP groups. TbTh increased significantly in 15% and 25% DP groups. Within the proximal tibia and when compared to controls: TbSp decreased significantly in 15% and 25% DP groups. When compared to controls: cortical thickness increased significantly in 5%, 15%, and 25% DP groups. 25% of DP significantly suppressed increase in urinary DPD excretion. 5%, 15%, and 25% DP groups significantly suppressed serum P1NP.Compared to controls: 15% and 25% DP groups significantly suppressed increase in cancellous BFR and MS/BS. 15% and 25% DP groups significantly suppressed increase in cancellous BFR and MS/BS. 15% DP groups significantly suppressed increase in MAR MS/bone area, BFR/BV, Periosteal BFR levels were significantly decreased in 5%, 15%, and 25% DP groups. All groups of DP increased significantly Bmp4 expression. 25% group significantly increased IGF-1 expression. Relative abundance of NFATc1 mRNA was significantly lower in all DP groups. No significant differences on other markers.
Smith et al. 2014a [29]	USA	Male C57BL/6 mice	Dried plum (*Prunus domestica*)	Osteopenic rats were placed on 25% DP intervention vs. a control diet.	Whole-body and vertebral (BMD, BMC, BMA) lumbar vertebra, distal femur, femur mid-diaphysis (BV/TV, TbN, TbTh, TbS, connective density, SMI), P1NP, PYD, glutathione peroxidase activity, OcS, ObS, MS, BFR, MAR, Pparc, Osx, Bmp2, Bmp4, ALP, Col1a1, OC, RANKL, OPG, NFATc1, and Ctsk.	4 or 12 weeks	At 4weeks: Whole body BMD, vertebra (BMD, BMC) were significantly higher in the DP group. Lumbar BV/TV, connective density, femur cortical thickness, were significantly higher in the DP group. SMI was significantly lower in the DP group. At 12 weeks: Whole body BMA, BMC and BMD, as well as vertebra BMD and BMC were significantly higher in the DP group. BV/TV (lumbar and distal femur), trabecular number (lumbar and distal femur), connectivity density (lumbar and distal femur), and femur cortical thickness, were significantly higher in the DP group. Trabecular separation (lumbar and distal femur), SMI (lumbar and distal femur) were significantly lower in the DP group. At 4 and 12 weeks, serum P1NP of DP group were significantly reduced compared to controls. At 12 weeks, serum PYD was significantly lower in the DP group. The DP group had significantly higher glutathione peroxidase activity than the control at 12 weeks. At 4 weeks, the DP group had significantly lower OcS, ObS, MS, and BMR. At 4 weeks, the DP group had significantly higher Pparc; lower Osx, Bmp4, ALP, Col1a1, Bglap2, RANKL, and NFATc1.
Arjmandi et al. 2001 [30]	USA	Female Sprague-Dawley rats	(*Prunus domestica*)	Female rats were either ovariectomized or sham operated. The ovariectomized groups were then fed either a 5% or 25% DP supplemented diet vs. a control diet.	Trabecular (total area, bone area, % bone area). Cortical (total area, bone area, marrow space, endosteal perimeter, and periosteal perimeter.	45 days	Compared to the controls the 25% DP group had significantly higher trabecular BA. Unreported results (data not shown): DP diets dose-dependently enhanced IGF-1.

ALP = alkaline phosphatase; BAK1 = BRI1-associated kinase 1; BFR = bone formation rate; BMA = bone mineral area; BMC = bone mineral content; BMD = bone mineral density; BMP2 = bone morphogenetic protein-2; BMP4 = bone morphogenetic protein-4; BS = bone surface; BS = bone surface; BV = bone volume; Casp3 = caspase-3; Casp9 = caspase-9; Col1a1 = collagen type 1a1; Coll1a = collagen type 1; CPII = C-propeptide of type II collagen; Ctsk = cathepsin K; CTX = C-terminal telopeptide of type II collagen; DP = dried plum; DPD = deoxypyridinoline; DPP = dried plum polyphenols; FOS = fructooligosaccharides; IGF = insulin-like growth factor; IGF-1 = Insulin-like growth factor-1; IL-6 = Interlukin-6; LinAtt = Linear X-ray attenuation coefficient; MAR = Mineral absorption rate; MCP-1 = Monocyte chemoattractant 1; MS = Mineralizing surface; NFATc = Nuclear factor of activated T cells; NFATc1 = Nuclear factor of activated T cells-1; Nfe212 = Nuclear factor erythroid derived 212; NTx = N-telopeptides of type-1 collagen; ObS = Osteoblast surface; OC = osteocalcin; OcS = Osteoclast surface; OPG = osteoprotegerin; Osx = osterix; PINP = procollagen type I N-terminal propeptide; Pparc = proliferator-activated receptor gamma; PYD = pyridinoline; RANKL = receptor activator of nuclear factor kappa-B ligand; Runx2 = Runt-related protein 2; SMI = structural model indexTbN = trabecular bone number; TbSp = trabecular bone separation; TbTh = trabecular thickness; TNF-a = tumor necrosis factor-alpha; TRAP = tartrate-resistant acid phosphatase; TV = trabecular volume.

**Table 5:** Cell studies.

Reference	Location	Cell Type	Plum Product	Dose	Methods	Markers Measured	Results
Bu et al. 2008 [31]	USA	RAW 264.7 murine macrophage cells	DPE (*P. domestica*)	0, 10, 20, or 30 µg/mL dried plum polyphenols	Cells were cultured and treated with various doses of dried plum extract.	Osteoclast differentiation and activity.	DPE suppressed osteoclast differentiation and activity under normal, oxidative stress, and inflammatory conditions.
Bu et al. 2009 [32]	USA	MC3T3-E1 cells	DPP (*P. domestica*)	0, 2.5, 5, 10 and 20 μg/mL	Cells were plated and pretreated with dried plum extracts and later stimulated with TNF-α.	Osteoblast function, mineralized nodule formation, and ALP.	DPP significantly increased intracellular ALP activity under normal conditions and significantly restored the TNF-α-induced suppression of intracellular ALP activity. DPP increased mineralized nodule formation under normal and inflammatory conditions. DPP increased osteoblast activity and function.
Hooshmand et al. 2015 [33]	USA	RAW 264.7 cells	DPP (*Prunus domestica* L.)	0, 0.1, 1, 10, 100, 1000 μg/mL DPP	Cells were treated to different doses of DPP.	NO, COX-2, and MA	In comparison to LPS-treated control cells: 1000 μg/mL DPP significantly reduced NO production. 100 and 1000 μg/mL DPP significantly decreased reduced protein level of COX-2. 1000 μg/mL DPP significantly prevented oxidation-induced increase in MA level.

ALP = alkaline phosphatase; COX-2 = cyclooxygenase-1; DPE = dried plum extract; DPP = dried plum polyphenols; MA = malondialdehyde; NO = nitric oxide; TNF-a = tumor necrosis factor-alpha.
